# Extreme thrombocytosis in systemic juvenile idiopathic arthritis. A case report

**DOI:** 10.1186/s13052-019-0664-4

**Published:** 2019-06-24

**Authors:** Alessandra Iacono, Monica Sprocati, Anna Lisa Giuliani, Francesco Di Virgilio, Caterina Borgna-Pignatti, Giuseppe Maggiore

**Affiliations:** 1 Department of Medical Sciences, Pediatrics, University of Ferrara, Ferrara, Italy; 2 Department of Morphology, Surgery and Experimental Medicine, Section of Pathology, Oncology and Experimental Medicine, University of Ferrara, 44121 Cona, Ferrara, Italy

**Keywords:** Systemic arthritis, Thrombocytosis, Case report

## Abstract

**Background:**

Systemic onset juvenile idiopathic arthritis (SoJIA) is a rare inflammatory disorder characterized by remitting fevers, evanescent rash, generalized lymphadenopathy, hepatomegaly/splenomegaly, and/or serositis.

**Case presentation:**

Here we report the case of a 5 years-old girl with SoJIA complicated by severe thrombocytosis. Treatment with the Interleukin-1β (IL-1β) receptor antagonist Anakinra caused a fast reduction of blood platelets and of the associated systemic inflammatory response. Measurement of IL-1β, IL-6 and Tpo plasma levels at different time points confirmed the etiopathogenetic role of IL-1β in causing the thrombocytosis, while Tpo did not appear to be involved and this explains the excellent response to treatment with Anakinra.

**Conclusion:**

The excellent response to treatment with the IL-1β receptor antagonist, suggests a key pathogenic role of IL-1β in thrombocytosis as well as in the associated systemic symptoms of inflammation.

## Background

The case describes an Italian child affected by systemic juvenile idiopathic arthritis (SJIA) with severe thrombocytosis (maximum value 3,193 platelets × 10^3^/μl). At our knowledge, a similar case was not present in literature.

In the attempt to explain the extremely high levels of platelets, we conducted an assay of IL-1β, IL-6 and Thrombopoietin (Tpo). The results of these measurements confirmed the etiopathogenetic role of IL-1β in causing the thrombocytosis, while Tpo did not appear to be involved. In fact, the child presented an excellent response to treatment with the IL-1β receptor antagonist.

## Case presentation

The patient was a 5 years old Italian girl in usual good health, regularly vaccinated. Family history was unremarkable. She was admitted at the emergency room of the Ferrara’s Hospital because of pain in her feet started 3 days before after a minor trauma, and of an abnormal gait with lower limbs extended.

The physical examination revealed the presence of ecchymosis at the ankles, and reduced mobility of the left tibiotarsic joint. No additional clinically relevant findings were observed on physical examination. The child was in good health, exception made for a feverish gastroenteritis 3–4 weeks before admission to the emergency room. She was discharged with anti-inflammatory therapy (Ibuprofen) every 8 h for 5 days, and recommending rest. However, once at home, she complained of night-time articular pain localized to the feet, and later extended to dorsal spine, hands and wrists. Wrists also showed ecchymosis. Fever was also present (maximum temperature of 38.9 °C).

Due to rapid deterioration of her conditions, the child was again referred to our emergency room. At the time of admission, she had an axillary temperature of 38.9 °C, aching and walking with lower limbs extended. Physical examination was suggestive of a polyarticular arthritis: joint pain elicited only by touching with bilateral functional limitation of tibiotarsic joints, knees, wrists, elbows and left metacarpophalangeal joints and in flexion the left hand-interphalangeal joints.

Pain was also triggered by cervical and dorsal spine movement. Cardio-respiratory objectivity, as well as pharynx and mucous membranes appeared normal, and no adenomegaly or hepatosplenomegaly was observed. The skin was normal, without nodules or rash.

Blood tests revealed high grade leucocytosis (leukocyte count 19,070/μL, 82.4% neutrophils), predominantly neutrophilia, with elevated inflammation indexes (ESR 56 mm, CRP 130.5 mg/l). The fast pharyngeal swab for haemolytic beta-streptococcus of Group A was negative. It was decided to admit the patient at the Pediatrics Department, where, while waiting for culture tests, we started intravenous antibiotic treatment with Amoxicillin and anti-inflammatory therapy with Ibuprofen every 8 h. This therapy produced a partial clinical recovery.

Further laboratory tests revealed Antinuclear Antibodies positivity (1:160, dotted score), Rheumatic Factor negativity, and confirmed the increased level of markers of inflammation: leucocytosis with neutrophilia (leukocyte count 20,030/μL, 82% neutrophils), thrombocytosis (772 platelets × 10^3^/μl), and increased ESR, CRP, alpha2globulin and ferritin (ESR 65 mm, CRP 164 mg/l, alpha2globulin 17.8%, ferritin 267 ng/ml). Pharyngeal swab for haemolytic beta-streptococcus was negative, and the anti-streptolytic title slightly increased (437 U/ml).

The electrocardiogram was normal, while the echocardiogram was suggestive of serositis: pericardial effusion in the absence of signs of hemodynamic repercussion. Abdominal ultrasound scan was normal and slit lamp examination showed no signs of eye inflammation. Thus, a tentative diagnosis of systemic inflammatory disease was made.

Therefore Kawasaki disease, rheumatic disease, streptococcal infection as differential diagnoses were excluded. Lymphoproliferative diseases were excluded by light microscope examination of peripheral blood and bone marrow aspirate smears.

Fever was intermittent with evening spikes. Intermittent fever, polyarthritis with poor response to anti-inflammatory drugs and serositis were suggestive of systemic juvenile idiopathic arthritis (SJIA).

First-line treatment with oral prednisone at the dose of 2 mg/kg/day induced a stable defervescence after 24 h (10th day of fever), and regression of arthritis.

Due to the marked thrombocytosis (1,515 platelets × 10^3^/μl), although ascribable to the inflammatory process, in addition to the corticosteroid treatment, we started antiplatelet therapy with aspirin at the dose of 5 mg/kg. During the hospitalization no clinical or laboratory evidence of macrophage activation syndrome was observed.

After about 2 weeks, the child was sent home in good general conditions with no articular and district signs of disease, except a residual minimal pericardial discharge. At that time, the platelet count was increased (2,597 × 10^3^/μl) in the absence of other abnormal laboratory indexes.

However, at follow-up visit 1 week after Hospital discharge, severe thrombocytosis (up to 3,193 platelets × 10^3^/μl) was found. Subcutaneous therapy with interleukin 1β (IL-1β) receptor antagonist (Anakinra 2.5 mg / Kg/ day) was started for a total of 22 administrations. IL-1β, interleukin-6 (IL-6) and thrombopoietin (Tpo) were measured before, throughout and after Anakinra treatment.

Platelet number decreased rapidly, with normalization achieved after the tenth dose (404 platelets × 10^3^/μl), with subsequent resolution of the pericardial effusion without coronary and valve involvement.

Residual blood samples after routine blood counts were used for plasma separation. Plasma aliquots were stored at − 80 °C avoiding freezing and thawing cycles. Plasma IL-6, IL-1β and Tpo levels were measured by using the Human IL-6 Quantikine, human IL-1β/IL-1F2 Quantikine, and the Human Thrombopoietin Quantikine ELISA kits, respectively (all from R&D System, Minneapolis, MN, USA), following manufacturer’s instructions. Samples were analyzed in duplicate. Optical density was measured with a Multiskan FC spectrophotometer (Thermo Scientific, Waltham, MA, USA).

Plasma samples were obtained at the following time points: the day before starting Anakinra treatment (T0), at day 14 (T1) after beginning treatment, at day 22 (the last day of treatment, T2), and after 10 months (T3). Plasma IL-6 levels (3.34 pg/ml at T0 and 3.92 pg/ml at T2, Table 1) were unaltered compared to normal values (12–13 pg/ml) [1], suggesting that IL-6 was not implicated in the disease process. On the contrary, plasma IL-1β levels at T0 were significantly increased (66.51 pg/ml). IL-1β levels were further increased at T1 (104.22 pg/ml), but drastically declined at T2 (17.75 pg/ml). At time T3, IL-1β concentration was 13.16 pg/ml, i.e. in the same range of concentrations reported for healthy children in other studies (8.1–19 pg/ml) [2] [3].

**Table 1:** IL-6, IL-1β, Tpo plasma levels and platelet number at the different time points.

Patient’s withdrawals	IL-6	IL-1β	Tpo	Platelet number
	pg/ml	pg/ml	pg/ml	
T_0_	3.34 (3.09-3.59)	66.51 (57.97-75.06)	51.88 (49.38-54.37)	3,193 x10^3^/μl
T_1_ (14^th^ day)	/	104.22 (102.69-105.75)	70.01 (63.13-76.87)	246 x10^3^/μl
T_2_ (22^nd^ day)	3.92 (4.08-3.75)	17.75 (16.76-19.08)	43.75 (41.87-45.63)	301 x10^3^/μl
T_3_ (10 months)	/	13.16 (7.56-18.76)	46.38 (41.38-51.37)	432 x10^3^/μl

Data are means of duplicates. The two values are reported in brackets

Finally, plasma Tpo levels were also examined at all time-points. Values were consistent with those reported for healthy adults (62.3 to 124 pg/ml [4]) and children (7 to 99 pg/ml [5]), and no significant changes were observed throughout the test period (Table 1). Due to limited availability of blood, it was not possible to measure all cytokines at all time points.

**Fig. 1:**
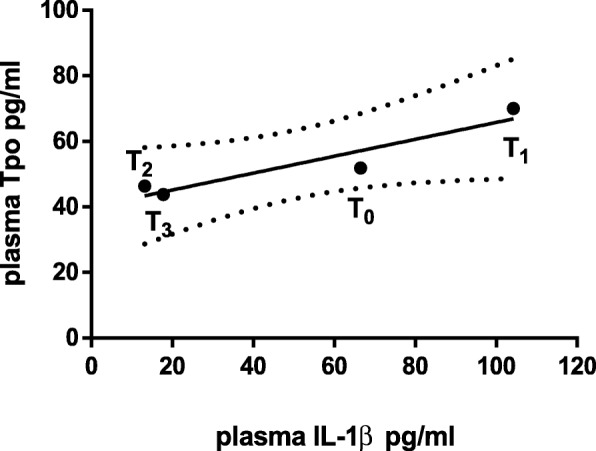
Correlation between IL-1β and Tpo plasma levels. The graph shows the correlation between IL-1β and Tpo plasma levels at the different time points (T1, 14th day, T2, 22nd day, T3, 10 months). The correlation was not statistically significant (R = 0.887, p = 0.057)

A correlation between IL-1β and Tpo plasma levels was tentatively verified (Fig. 1), but although the two parameters seemed to be correlated, statistical significance was not achieved (R = 0.887, *p* = 0.057).

Therapy with Anakinra was well tolerated, the only side effect observed being a skin rash at the injection sites that resolved rapidly and spontaneously. At four and 10 months follow-up, the child has normal platelet count (439 platelets × 10^3^/μl) and echocardiography test.

## Discussion

Systemic Juvenile Idiopathic Arthritis (SJIA) is clinically defined by daily fever (for at least 3 consecutive days) over 2 weeks or more, arthritis, and by at least one of the following symptoms: evanescent rash, generalized lymphadenopathy, hepatomegaly and/or splenomegaly or serositis. As SJIA is a diagnosis of exclusion, it excludes infection and malignancy [6] [7]. The epidemiology of SJIA features an early incidence peak between 2 and 6 years of age and accounts for approximately 11–20% of all types of Juvenile idiopathic arthritis [8]. First line treatment includes non-steroidal anti-inflammatory drugs (NSAIDs). Most commonly, symptoms of systemic inflammation persist or respond only incompletely. If there is no response to NSAIDs, oral systemic corticosteroids are started. Subsequently, therapies with biological disease-modifying anti-rheumatic drugs (bDMARDs) are administered: inhibitors of IL-6 (Tocilizumab), IL-1β (Canakinumab) or IL-1β receptor (Anakinra) [9].

The inflammatory process of SJIA primarily affects T cells whose activation involves a cascade of responses including B cells activation, consumption of complement factors and release of IL-6, IL-1β, Tumour necrosis factor α (TNF-α), and other proinflammatory cytokines. These factors play a key role in the autoimmune inflammation leading to damage at the joints and other affected tissues. Inflammatory cytokines also play a key role in platelet activation. On the basis of the established role of IL-1β in SJIA pathogenesis [10], and the diagnostic hypothesis that the increased platelet number and the pericardial effusion were signs of systemic involvement, we started a treatment with Anakinra that brought about a dramatic improvement in the patient’s overall clinical conditions. In addition, the platelet number, extremely high before starting treatment, declined to 404 platelets × 10^3^/μl after the 10 days of Anakinra administration.

IL-6 plasma levels did not change during the test period: T0 (3.92 pg/ml) and T1 (3.34 pg/ml). These values did not differ from those described in the literature [1], suggesting that an involvement of IL-6 could be excluded. On the contrary, the patient’s IL-1β plasma levels were substantially increased at T0 (66.51 pg/ml), even more so at T1 (104.22 pg/ml), and declined at T2 (17.75 pg/ml). At 10 months follow-up (T3), IL-1β plasma levels (13.16 pg/ml) were in the range reported for healthy adults and children [2, 3]. The IL-1β increase after 14 days of Anakinra treatment (T1) might be explained by the fact that the drug, engaging the receptor, had reduced its availability to bind the natural agonist, i.e. IL-1β. The IL-1β value returned to normal after 22 days of treatment and remained unchanged after 10 months.

The platelet number was rapidly reduced to normal values (246 platelets × 10^3^/μl) by Anakinra. Because thrombocytosis might also be due to increased Tpo secretion, we also measured Tpo plasma levels. Tpo is a hormone produced by the liver and kidneys that can induce platelet production and maturation by acting on megakaryocyte receptors. A relationship between increased IL-β and Tpo levels was previously shown [11]. Tpo value, which was 51.88 pg/ml at T0, slightly increased to 70.01 pg/ml at T1 and decreased to 43.75 pg/ml at T2. At T3, Tpo level was 46.38 pg/ml. These values were very similar to those reported for healthy children [4] and adults [5], and much lower than values found in adult subjects with thrombocytopenia (476–3,148 pg/ml) (our unpublished data).

Changes in IL-β and Tpo plasma levels showed a close albeit non statistically significant correlation (Fig. 1).

## Conclusions

The clinical case of our patient is interesting for the extreme thrombocytosis, to the best of our knowledge never previously reported in the literature, and for its excellent response to treatment with the IL-1β receptor antagonist, suggesting a key pathogenic role of IL-1β in thrombocytosis as well as in the associated systemic symptoms of inflammation.

## Data Availability

All data generated or analysed during this study are included in this published article.

## Abbreviations

CRP: C-reactive protein
ESR: Erythrocyte sedimentation rate
IL-1β: Interleukin-1β
IL-6: Interleukin-6
SJIA: Systemic juvenile idiopathic arthritis
Tpo: Thrombopoietin
